# Olecranon Fractures Managed Using FiberWire Sutures Without Metallic Implants: A Case Study and a Review of the Literature

**DOI:** 10.7759/cureus.77336

**Published:** 2025-01-12

**Authors:** Abdulrahman Alaseem, Rheema Alfadhil, Hayfaa Alshaalan, Sarah AlQuwayz, Sarah Alaidarous, Banan Alqady, Alwateen Albalawi, Sarah Alflaij, Muhammad Asif

**Affiliations:** 1 Orthopaedic Surgery, College of Medicine, King Saud University, Riyadh, SAU; 2 Orthopaedic Surgery, McGill University, Montreal, CAN; 3 Orthopaedic Surgery, Ministry of Health Holdings, Riyadh, SAU

**Keywords:** fiberwire suture, kirschner wires, olecranon fracture, olecranon repair, transosseous sutures

## Abstract

The traditional standard of care, tension band wire fixation modalities commonly used to treat non-comminuted olecranon fractures, are frequently associated with complications, with symptomatic hardware being the most common issue, often necessitating subsequent surgical procedures for hardware removal. We present a case of a young, active gentleman who sustained a simple olecranon fracture (Mayo type IIA) and underwent open reduction with the innovative all-suture non-metallic internal fixation procedure. We used a low-profile, cost-effective alternative fracture fixation technique, following the principles of pre-existing surgical techniques, while utilizing FiberWire sutures with biomechanical properties equivalent to, or possibly superior to, conventionally used metallic wires, thereby eliminating the need for additional surgical intervention. Furthermore, we reviewed the literature for similar reported cases and highlighted their results and outcomes.

## Introduction

Olecranon fractures make up 40% of all fractures occurring around the elbow and constitute 10% of fractures in the upper extremity [1-3]. The vast majority of olecranon fractures require surgical intervention. The most commonly used fixation techniques in olecranon fractures are tension band wiring (TBW), intramedullary screws, or plate fixation [4-6]. Although all yield excellent clinical outcomes, many postoperative complications have been reported due to the subcutaneous location of the olecranon. These include soft tissue irritation, nonunion/malunion, motion restriction, infections, ulnar palsy, or symptomatic hardware that may lead to subsequent implant removal [3,6,7].

Transosseous sutures were proposed as an alternative solution to overcome some complications associated with metallic implants. They are considered a reliable fixation method and have lower reoperation rates than other techniques [8-11]. This method is regarded as a safe, cost-effective, and good alternative to traditional TBW, reducing the rate of symptomatic hardware or other complications associated with conventional techniques for olecranon fractures [8,11,12].

In the following paper, we report a case in which a simple transverse olecranon fracture was fixed using FiberWire sutures only in a modified pretzel knot technique without the placement of any supporting metallic implants.

## Case presentation

A 31-year-old medically free gentleman presented to the emergency department after falling from his bicycle while cycling downhill. The patient fell onto his chest and right elbow, trying to avoid falling onto his head. He had no other injuries and was not wearing a helmet. 

**Figure 1:**
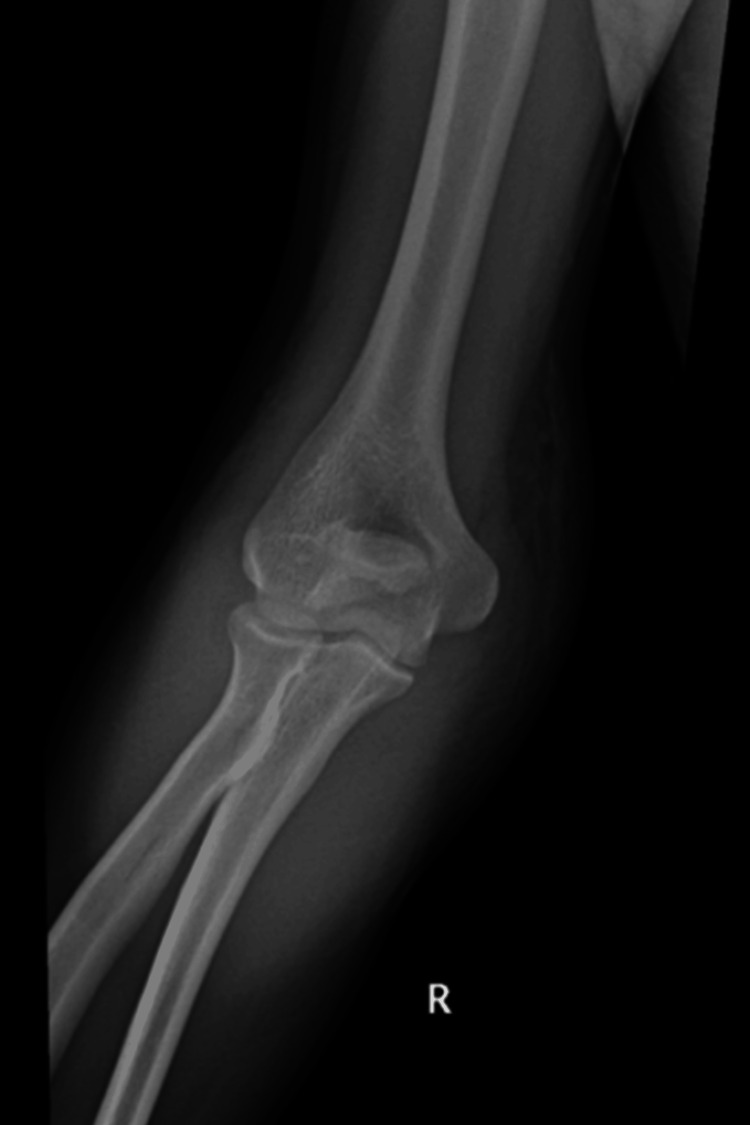
Preoperative AP X-ray image of the right elbow. AP, anteroposterior

**Figure 2:**
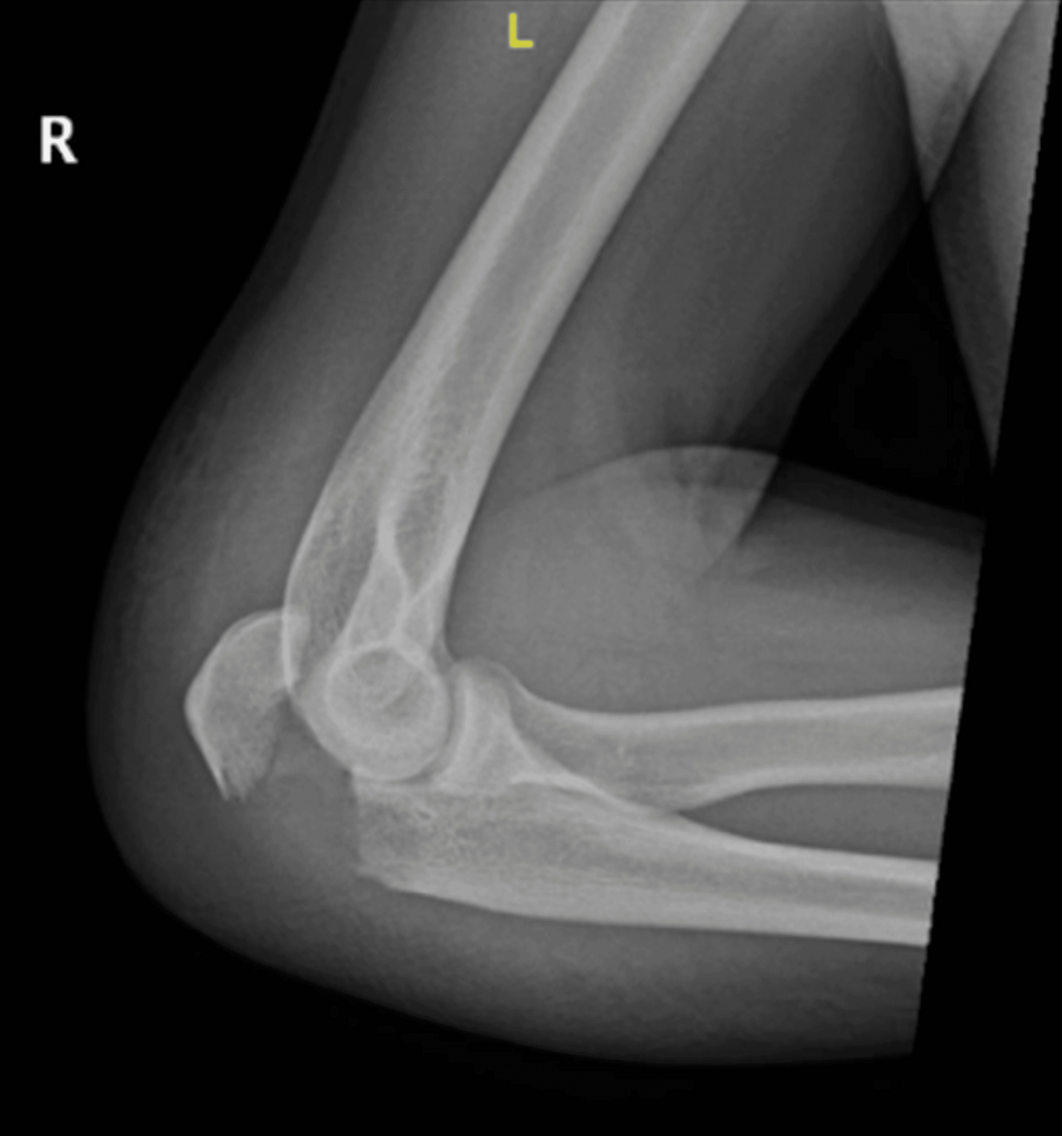
Preoperative lateral X-ray image of the right elbow.

Upon physical examination, the patient was alert and responsive. The local examination revealed an obvious deformity of the right elbow, moderate swelling, and tenderness over the elbow with limited range of motion (ROM). The compartments were soft, and the distal neurovascular status was intact. His X-ray revealed a displaced transverse/short oblique non-comminuted olecranon fracture (Mayo type IIA, Colton II, AO 21B1) (Figure 1 and Figure 2). The patient was booked for right olecranon fracture open reduction and internal fixation (ORIF). If feasible, he requested to avoid any metallic implant, as he wishes to prevent any further surgical procedure for hardware removal.

After prepping and draping the patient in a supine position, a skin incision was made over the right ulna, curving around the elbow. Subcutaneous dissection was carried out to expose the fracture site. After refreshing the fracture edges, we examined the fracture pattern again. It was a transverse/short oblique minimally comminuted fracture on the medial side.

**Figure 3:**
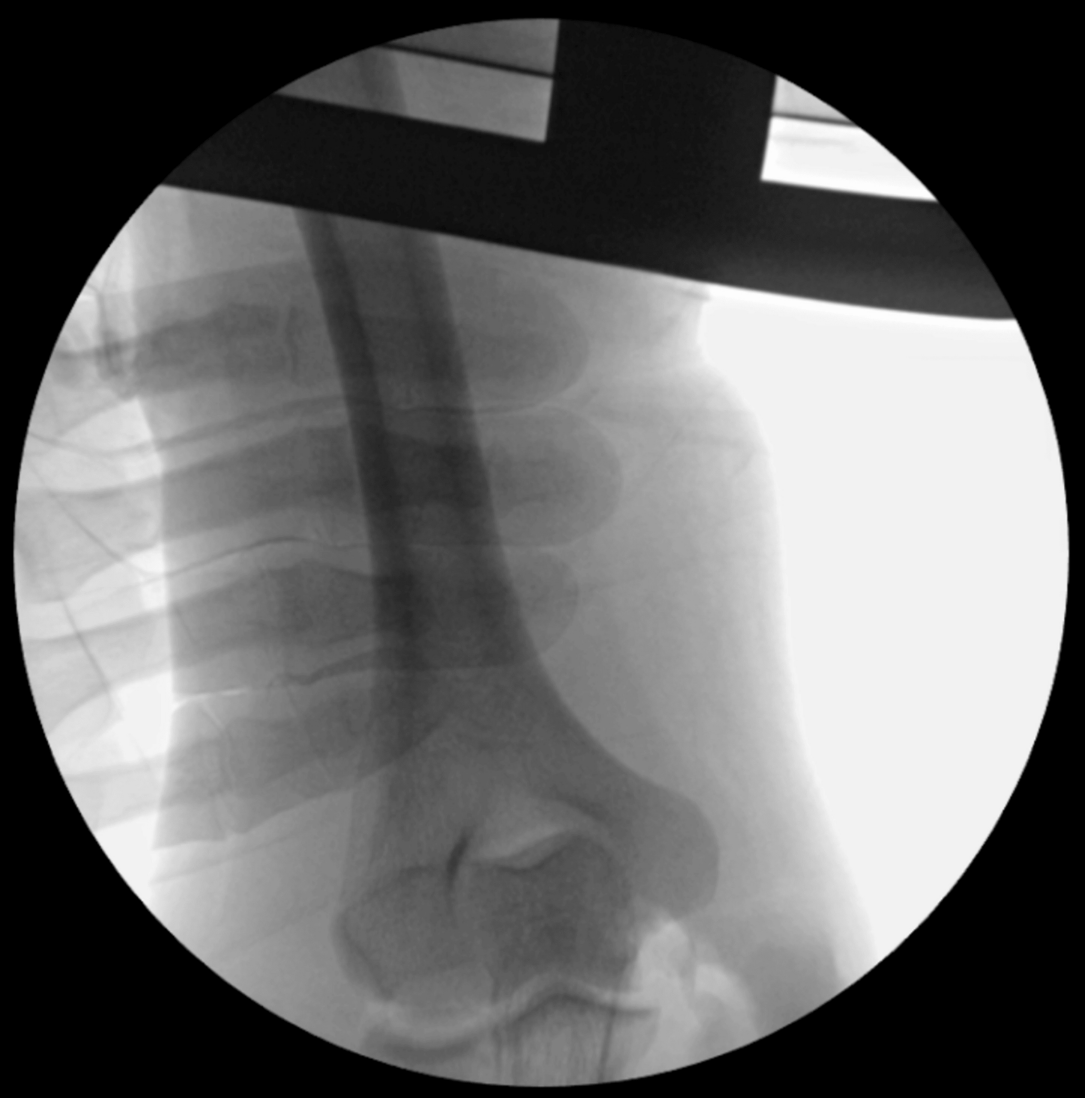
Intraoperative AP X-ray image (fluoroscopy) of the right elbow. AP, anteroposterior

**Figure 4:**
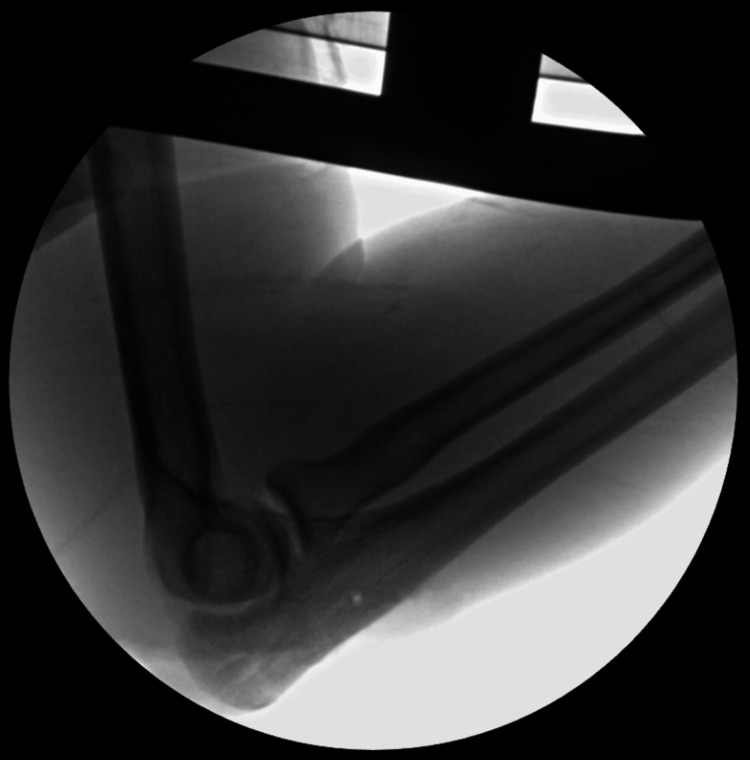
Intraoperative lateral X-ray image (fluoroscopy) of the right elbow.

Two Kirschner wires (K-wires) were used to temporarily hold the fracture site in a reduced position after X-ray confirmation of reduction. Fixation was achieved using two No. 2 FiberWire sutures, tied in a pretzel/square knot technique, creating a tension band construct. Two sutures were tied and tightened with the elbow in flexion; then, another two sutures were pulled with the elbow extended to ensure optimal compression over the fracture site. The K-wires were subsequently removed. Final intraoperative X-ray imaging and clinical assessment confirmed joint congruency, fracture compression, and the absence of fracture gaping (Figure 3 and Figure 4). The wound was copiously irrigated, and the closure was performed in layers. A sterile dressing and backslap were applied.

Postoperatively, the patient was discharged the following day after receiving prophylactic antibiotics. His pain was effectively managed with analgesics, and his distal neurovascular status was intact with soft compartments. 

**Figure 5:**
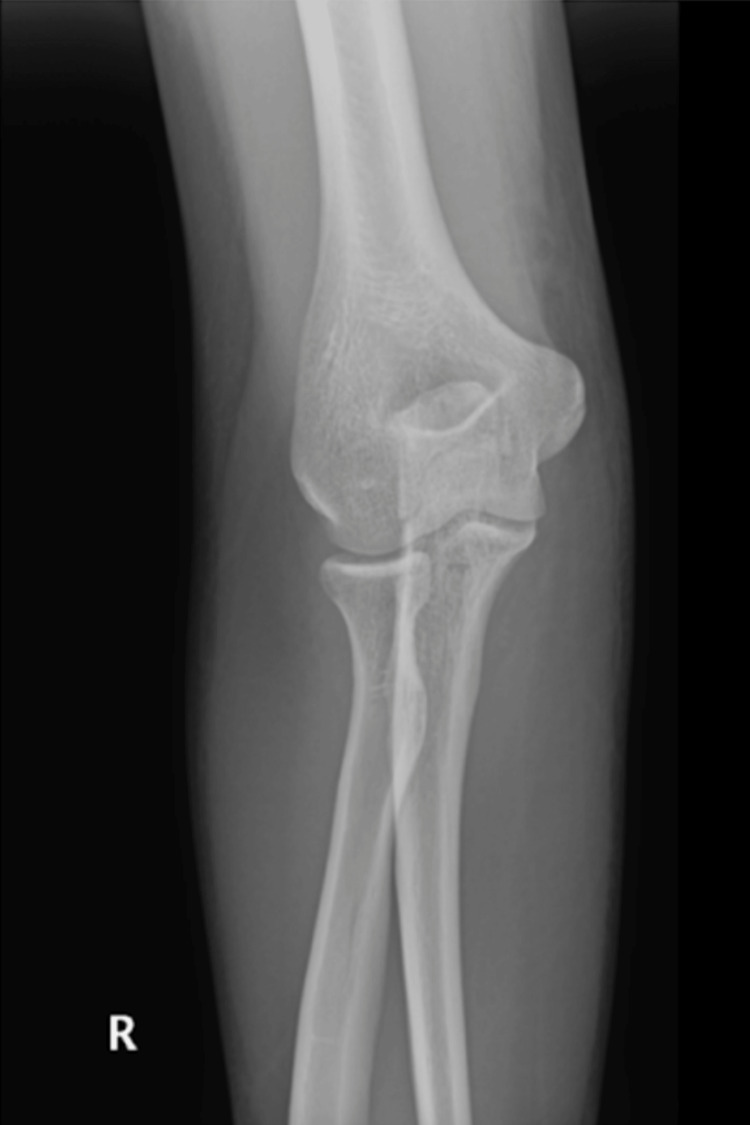
Six weeks postoperative AP X-ray image of the right elbow. AP, anteroposterior

**Figure 6:**
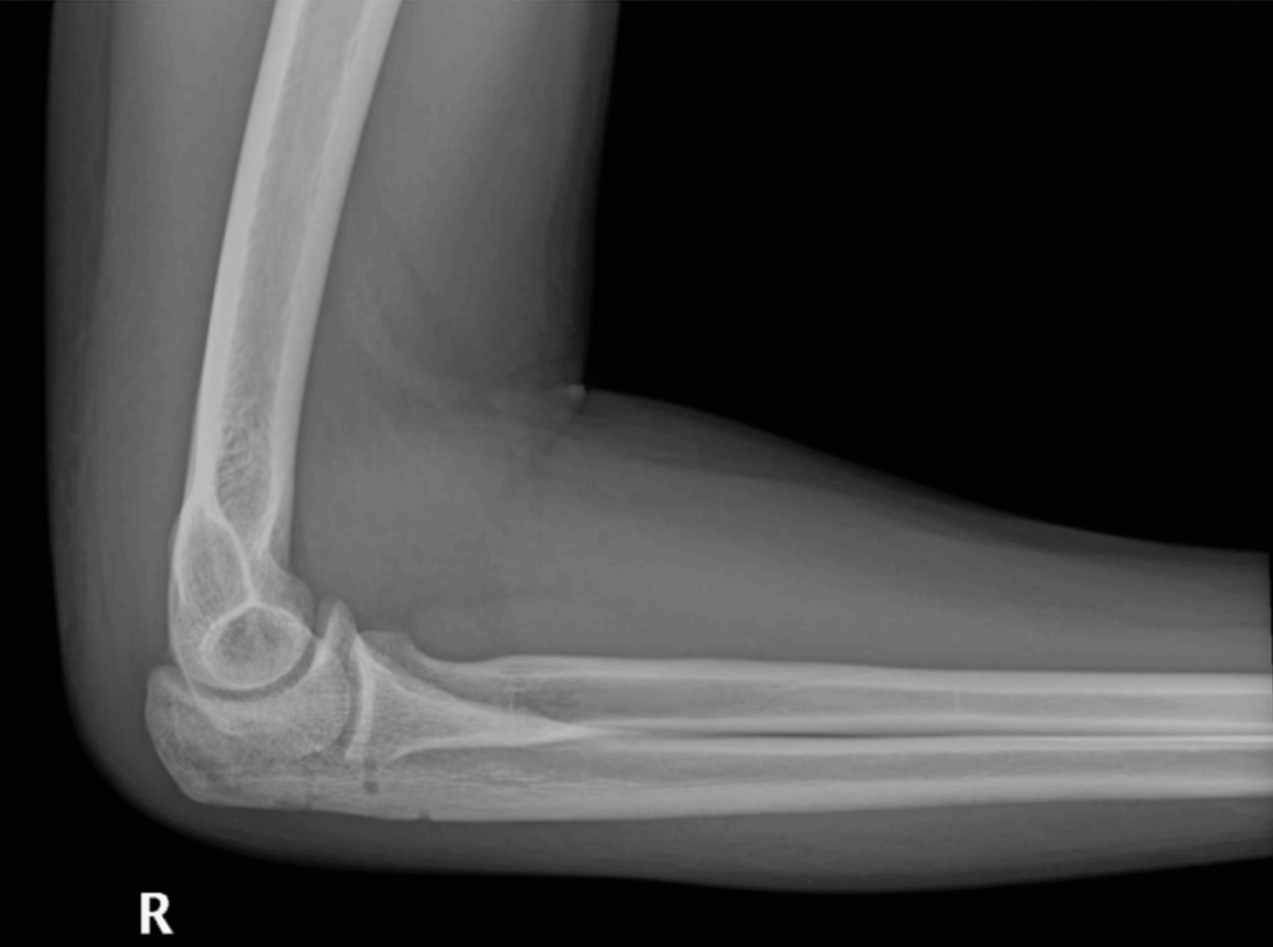
Six weeks postoperative lateral X-ray image of the right elbow.

At two weeks' follow-up, the sutures and backslap were removed, and the patient began gentle passive elbow ROM exercises. Six weeks postoperatively, the patient demonstrated an almost full ROM and his X-rays showed good consolidation at the fracture site with no evidence of gaping (Figure 5 and Figure 6). The patient was periodically followed up every three months until one year postoperatively. He was provided with a gradual rehabilitation protocol to regain his elbow ROM and strength, which he was compliant with, and he achieved optimal outcomes.

## Discussion

Olecranon non-comminuted fracture fixation with traditional TBW poses a high risk of symptomatic hardware and frequently requires hardware removal, which carries a substantial quality of life and economic burden both to patients and the health care system. The all-suture olecranon fixation technique we presented with the modifications reported in the literature combines the principles of TBW. Nevertheless, it avoids the discomfort associated with symptomatic metallic hardware and the cost of reoperation to remove symptomatic hardware. Utilizing the biomechanically strong FiberWire sutures and bone tunnels, we provide a safe, effective, low-profile, cost-effective with minimal discomfort, and reproducible alternative to conventional standard-of-care surgical fixation techniques, such as TBW. A recently published systematic review addressing high-strength sutures and anchors in olecranon fractures showed a mean age of 66 years within 131 participants across nine inclusion-qualified studies. The article reviewed several studies that found that high-strength suture techniques for tension banding and anchoring effectively maintained stability in displaced olecranon fractures. This approach also led to fewer complication rates and a low rate of reoperations. Suture structures offered substantial economic benefits, primarily by eliminating the necessity for further implant removal. They revealed that tension band suturing for displaced olecranon fractures might be a viable and more economical alternative to traditional band wiring. This approach could help ensure adequate fixation, decrease hardware-related complications, and reduce the need for revision surgeries [13].

Nimura et al. reported two cases of olecranon fractures managed using FiberWire sutures without the need for metallic implants. As in our case, two K-wires were placed from the dorsal cortex of the distal segment to the tip of the olecranon after reducing the fracture fragments. However, unlike our approach, which involved a suture retriever, they used K-wires instead. They passed two strands of FiberWire twice, manually tensioning and knotting them on the posterior surface of the olecranon. One patient experienced a minor reduction in flexion and extension compared to the opposite elbow. Despite this, both cases resulted in successful bony union without reports of pain or skin irritation [12].

Henseler et al. also described their technique for suture tension banding done on three elderly patients with displaced olecranon fractures, and all three patients achieved good outcomes. According to the AO classification, the patients with olecranon fractures were categorized as 21A1 and 21B1. Furthermore, they were classified as type IIA in line with the Mayo system. Their patients were positioned supine, and the standard dorsal approach was employed. Once the olecranon fracture was stabilized, two holes, each measuring 2 mm, were made in the distal ulnar fragment, situated as adjacent to its anterior aspect as achievable. Additionally, two supplementary drill holes were created in the proximal fragment, ensuring they were positioned superior to the triceps insertion site to mitigate the risk of tendon injury. Ethibond No. 6 sutures were employed, with both ends threaded through the distal drill hole and guided into the intramedullary canal at the fracture site. They were then retrieved from the proximal ulna, just above where the triceps inserts. The sutures were subsequently secured using a simple non-sliding knot. Two more holes were created in the distal fragment, following the traditional approach of cerclage wiring. A second suture was passed through these holes in a figure-of-eight configuration and secured laterally at the triceps insertion to provide further compression to stabilize the fracture, mimicking polyester tension-band wiring’s function. A suture of FiberTape is used in case of a triceps cut-through incident, a multi-stranded, long polyethylene material recognized for its outstanding ability to resist tissue cut-through. In the case at hand/under discussion, the initial suture functioned like a K-wire, whereas the subsequent one served as a tension-band wire. Postoperatively, all achieved bone union by their 12-week visit. All achieved complete active extension and supination/pronation, whereas their flexion ranged from 120° to 130°. None had suture failure, malreduction, fracture displacement, ulnar neuropathy, or infection [3].

Phadnis et al. presented a similar surgical technique for treating Mayo IIA olecranon fractures. They described it as having the patient in the lateral decubitus position and using a direct posterior incision to reach the fracture site. A 2.5 mm transverse drill hole is created through the ulna distal to the fracture. Two sets of No. 2 braided non-absorbable sutures are utilized in the procedure. The first set is passed from lateral to medial through the drill hole to engage the medial triceps insertion on the proximal fragment, subsequently redirected from medial to lateral through the same drill hole to engage the lateral triceps insertion. The suture ends are tensioned and tied over the lateral aspect of the olecranon. The second set of sutures follows a similar pathway, initially passing laterally to medially through the drill hole, this time grasping the posterolateral triceps insertion. The suture is then passed back from medial to lateral through the drill hole to engage the posteromedial triceps insertion. These suture ends are likewise tensioned and tied laterally on the ulna, adjacent to the first suture. Once the reduction clamp is removed, a fluoroscopic examination is performed to check for gaps and measure the elbow's ROM [9,10].

Similarly, Das et al. described a case series of 10 patients. Three had chevron olecranon osteotomies done, and the rest had simple olecranon fractures. All the cases were treated with suture repair. This method involved utilizing a durable braided polyester and polyethylene suture passed through a tunnel in the bone. Initially, a transverse hole was drilled in the ulna with a 2.5 mm drill. Following this, fracture stabilization was accomplished by threading two synthetic braided sutures through the bone tunnel to anchor triceps tendon insertion. In their results, all their cases united clinically and radiologically at six weeks. Only one malunion was reported [11].

When comparing outcomes of transosseous suture fixation with plate fixation and TBW, surgical revision was found to be relatively higher, with TBW at 36% and plate fixation at 11%, compared to only 2% in patients treated with suture fixation [9]. Moreover, excellent results were reported in treating simple olecranon fractures and osteotomies using non-absorbable braided sutures without needing any metalwork, tunnels, or suture anchors [8,11].

Additionally, another study examined the biomechanical properties of tension bands compared to those made from 18-gauge metal wire. The research evaluated these tension bands when used alongside either intramedullary screws or K-wires to determine their effectiveness and strength. They concluded that they were equivalent to each other. They also concluded that the fatigue patterns exhibited by using high-strength suture tension bands were analogous to metal wire tension bands [14]. Contreras Fernandez et al. performed an observational retrospective study investigating the outcomes of using an intramedullary cancellous screw and suture tension band construct to treat Mayo type IIA olecranon fractures by using ROM and several outcome scoring systems. In their study, they utilized an intramedullary 6.5 mm AO cancellous screw and No. 2 FiberWire suture. They concluded that this surgical technique was excellent and associated with lower rates of complications and the need for subsequent material removal [15].

Our case is unique as we modified the pretzel (square knot) technique, incorporating two knots. One was tied in flexion, and the other in extension. Both were done using FiberWire sutures. Theoretically, the second knot may augment the first one, leading to a stronger fixation construct and avoiding any gap formation that may occur with elbow ROM. 

In addition, in our patient, we did not observe any triceps muscle compromise or reduction in strength. Furthermore, there was no evidence of improper alignment or shifting of the fracture. He regained full elbow ROM and extension strength when he came for a nine-month follow-up. Hensler et al. managed to follow up with their patients long enough to be able to report no experience of the complications [3].

The present study has several limitations. First, it is a report of a single case. Nonetheless, he has an excellent functional outcome in regaining full ROM and strength, initially with minimal discomfort due to slightly prominent suture knots. Moreover, he was delighted to enjoy playing guitar and not have any metalwork on his elbow. Neither did he have any complications, nor did he require any surgical intervention until one-year follow-up postoperatively. One concern regarding braided sutures is their capability to handle the considerable tensile forces generated by the triceps muscle. Nonetheless, laboratory experiments and studies on live animals have indicated that these sutures exhibit tensile strength and fatigue resistance similar to stainless steel wires [10].

## Conclusions

The use of non-absorbable high-strength polyethylene sutures (FiberWire sutures) could be considered a biomechanically equivalent, less symptomatic, and cost-effective alternative to the conventional tension-band wiring technique for simple displaced olecranon fractures. The patient in this study did not suffer postoperative complications and regained full ROM and strength, highlighting the efficacy of the used technique. Nonetheless, it must be utilized cautiously, with careful patient selection, to achieve desirable outcomes. We recommend that this technique be investigated further in large multicenter randomized control trials, comparing its results with those reported in the standard-of-care conventional fixation modalities. We also recommend investigating its use in the fixation of olecranon osteotomies. 
